# Supplementary site interactions are critical for the regulation of microsomal triglyceride transfer protein by microRNA-30c

**DOI:** 10.1186/1743-7075-10-56

**Published:** 2013-09-04

**Authors:** James Soh, M Mahmood Hussain

**Affiliations:** 1 Department of Cell Biology, SUNY Downstate Medical Center, Brooklyn, NY 11203, USA; 2 Department of Pediatrics, SUNY Downstate Medical Center, Brooklyn, NY 11203, USA; 3 VA New York Harbor Healthcare System, Brooklyn, NY 11209, USA

**Keywords:** MTTP, apoB, Lipoproteins, microRNA, miR-30, Hyperlipidemia, UTR

## Abstract

Microsomal triglyceride transfer protein (MTTP) is an essential chaperone that assists in the assembly of apolipoprotein B-containing lipoproteins to transport lipids. We have shown that microRNA (miR)-30c regulates MTTP expression but other members of the same family do not. Further, we showed that interactions between miR-30c seed sequence and the 3΄-untranslated region (UTR) of the MTTP mRNA are critical for this regulation. The same seed sequence is shared by all the members of the miR-30 family. Therefore, it is unclear why only miR-30c regulates MTTP expression. Bioinformatics analysis revealed that, miR-30c interacts with MTTP mRNA involving supplementary site, besides seed sequence, forming an intervening loop. Here, we evaluated the importance of the supplementary site and the size of the intervening loop in miR-30c/MTTP mRNA interactions by cloning *MTTP* 3΄-UTR at the end of the luciferase gene and subjecting it to site-directed mutagenesis. Reducing the number of base pairs at the supplementary site abolished the ability of miR-30c to reduce luciferase activity. However, increasing the number of base pairs at the supplementary site, seed sequence or in the intervening loop enhanced the efficacy of miR-30c in reducing luciferase activity. These studies demonstrated that the supplementary site of miR-30c is, but the intervening loop is not, critical for binding to the MTTP mRNA. To our knowledge, this is the first demonstration that miRs might require both seed and supplementary interactions to regulate target mRNA specificity. Further, this study suggests that more potent miR-30c mimics could be synthesized by increasing base pairing in the loop region.

## Introduction

The lipoprotein secretion pathway is controlled by two critical factors: MTTP and apolipoprotein B (apoB) [1]. MTTP serves as a chaperone in the proper assembly and secretion of apoB-containing lipoproteins. The significance of MTTP was initially revealed in a condition called abetalipoproteinemia [2]. These patients have null mutations in the *MTTP* gene and exhibit virtually no plasma apoB lipoproteins [3,4]. This discovery highlighted the importance of MTTP in the lipoprotein assembly and secretion pathway and suggested the possibility that its inhibition might be useful in reducing plasma lipids. Subsequently, several chemical inhibitors of MTTP have been developed. These antagonists successfully block MTTP activity and lower plasma lipids. However, they also cause hepatic steatosis and increase plasma transaminases [5,6]. Hence, attempts are underway to target MTTP while avoiding adverse effects associated with lowering plasma lipids. Several approaches have been proposed to inhibit MTTP while avoiding toxicity [7]. It has been suggested that toxicity associated with MTTP inhibition is mainly related to accumulation of lipids in the liver [8]. Therefore, a combined use of MTTP inhibition with agents that lower hepatic lipids might be useful. Further, intestine-specific MTTP antagonists have been designed to circumvent unwanted side effects associated with hepatic MTTP inhibition [9].

Recently, microRNAs (miRs) have emerged as novel therapeutic agents. For example, it has been shown that inhibition of miR-33 reduces atherosclerosis by increasing plasma HDL concentrations [10,11]. Similarly, miR-122 antagonism has been used as a possible therapeutic option in the treatment of Hepatitis C virus [12]. miRs are endogenous ~22 nucleotide long RNAs that interact with various mRNAs to modulate their translational efficiency and/or their mRNA stability thereby modulating the amounts of proteins synthesized. miRs interact with the 3΄-untranslated regions (UTRs) of several target mRNAs involving seed sequences. Since miRs interact with several mRNA, they affect different metabolic pathways. Hence, miRs provide a novel opportunity to regulate pathways instead of individual target proteins.

Using bioinformatics database, TargetScan, we found that the miR-30 family members could interact with the 3΄-UTR of MTTP approximately 89 bases away from the stop codon [13]. Furthermore, the interaction sites in both the MTTP 3΄-UTR and miR-30 family members were conserved through evolution among vertebrates. Further analyses revealed that one member of the family, miR-30c, reduced hepatic MTTP expression both *in vitro* and *in vivo*[13], while the other members of the miR-30 family did not reduce MTTP expression. Reductions in MTTP expression were associated with decreases in plasma lipids and atherosclerosis in different mouse models [13]. Here, we wanted to know why only miR-30c regulates MTTP. miR-30c seed sequence interacts with MTTP mRNA involving 6 nucleotide base pairs [13] and is considered “marginal” and not a “canonical” site for miR target interactions [14,15]. Marginal site is defined as having six perfect matches at the seed sequence; canonical site refers to at least seven perfect matches. Further, miR-30c has an atypical supplementary base pairing site involving seven base pairs. Based on this information, we hypothesized that the specificity of interactions between miR-30c and MTTP may be determined by specific supplementary interactions and the positioning of the loop between seed and supplementary sites. We evaluated this hypothesis by modulating 3΄-UTR sequences in the MTTP that interact with miR-30c.

## Materials and methods

### Plasmid construction

The entire 3΄-UTR (1149 bp) of human MTTP mRNA was amplified by PCR and cloned into psiCHECK2 (Promega) plasmid between *Not1* and *Xho1* restriction sites. Site-directed mutagenesis was performed using QuikChange II XL (Agilent Technologies) to produce different mutations. Primers used for PCR amplification and sequencing were designed using PrimerExpress 3.0. In addition, mutagenesis primers were designed using Agilent Technologies Primer Design software (https://www.genomics.agilent.com/) (Table 1). Sequence identity of all plasmid mutations were verified by Macrogen, Inc., sequencing services.

**Table 1:** Primers used for site-directed mutagenesis

	**Primers for mutagenesis**
MTTP_U75G_	Sense: 5′-tgactaagtacttgctcgctgagagcacagcgttt-3′
	Anti-sense: 5′-aaacgctgtgctctcagcgagcaagtacttagtca-3′
MTTP_U75G,C83U_	Sense: 5′-gcatgactaagtacttgctcgctgagagtacagcgtttacatatttacc-3′
	Anti-sense: 5′-ggtaaatatgtaaacgctgtactctcagcgagcaagtacttagtcatgc-3′
MTTP_G82A_	Sense: 5′-ctaagtacttgctctctgagaacacagcgtttacatatttacc-3′
	Anti-sense: 5′-ggtaaatatgtaaacgctgtgttctcagagagcaagtacttag-3′
MTTP_A81U,G82A_	Sense: 5′-gactaagtacttgctctctgagtacacagcgtttacatatttacct-3′
	Anti-sense: 5′-aggtaaatatgtaaacgctgtgtactcagagagcaagtacttagtc-3′
MTTP_G90U,C91A_	Sense: 5′-gctctctgagagcacagcatgtttacatatttacctgta-3′
	Anti-sense: 5′-tacaggtaaatatgtaaacatgctgtgctctcagagagc-3′
MTTP_C85U,C88G_	Sense: 5′-ctaagtacttgctctctgagagcatagggtttacatatttacctgtatttaa-3′
	Anti-sense: 5′-ttaaatacaggtaaatatgtaaaccctatgctctcagagagcaagtacttag-3′
MTTP_C83U,A84G,C85U_	Sense: 5′-catgactaagtacttgctctctgagagtgtagcgtttacatatttacctgtatttaa-3′
	Anti-sense: 5′-ttaaatacaggtaaatatgtaaacgctacactctcagagagcaagtacttagtcatg-3′

### Cell culture and transfection with plasmid DNA or miRs

COS-7 cells were cultured in DMEM supplemented with 10% FBS, 1% penicillin/streptomycin, amphotericin and 1% glutamine. For luciferase plasmid transfections, approximately 1.2 × 10^6^ COS-7 cells were plated in 100 mm tissue culture plates and reverse transfected with 50 nM Scr (5΄-UCACAACCUCCUAGAAAGAGUAGA) or miR-30c (5΄-UGUAAACAUCCUACACUCUCAGC) using RNAiMAX transfection reagent (Invitrogen). After ~ 17 hours, cells were transferred to 24-well tissue culture plates (5 × 10^5^ cells/well) and reverse transfected using Lipofectamine 2000 transfection reagent (Invitrogen) again with psiCHECK2 plasmid (1 μg/well) carrying either wildtype or mutant MTTP 3′-UTR sequences. Cells were incubated for another 17 hours and collected for analysis and measured for luciferase activity.

### Luciferase assay

Transfected cells were washed with PBS two times and incubated with 1× Passive Lysis Buffer (Promega). Incubation with Passive Lysis Buffer was done for approximately 30 minutes at room temperature. Luciferase activity was measured using a luminometer. LAR II and *Stop-n-Glo* substrates (Promega) were used for luciferase activity and *Renilla* luciferase activity, respectively.

### Statistical analysis

Experiments were performed in triplicates and repeated at least three times. Data are presented as mean ± standard deviation. Statistical significance (p < 0.05) was determined using one-way analysis of variance (One-Way ANOVA) with GraphPad Prism software.

## Results

### Predicted interactions between miR-30 family members and *MTTP* mRNA

**Figure 1:**
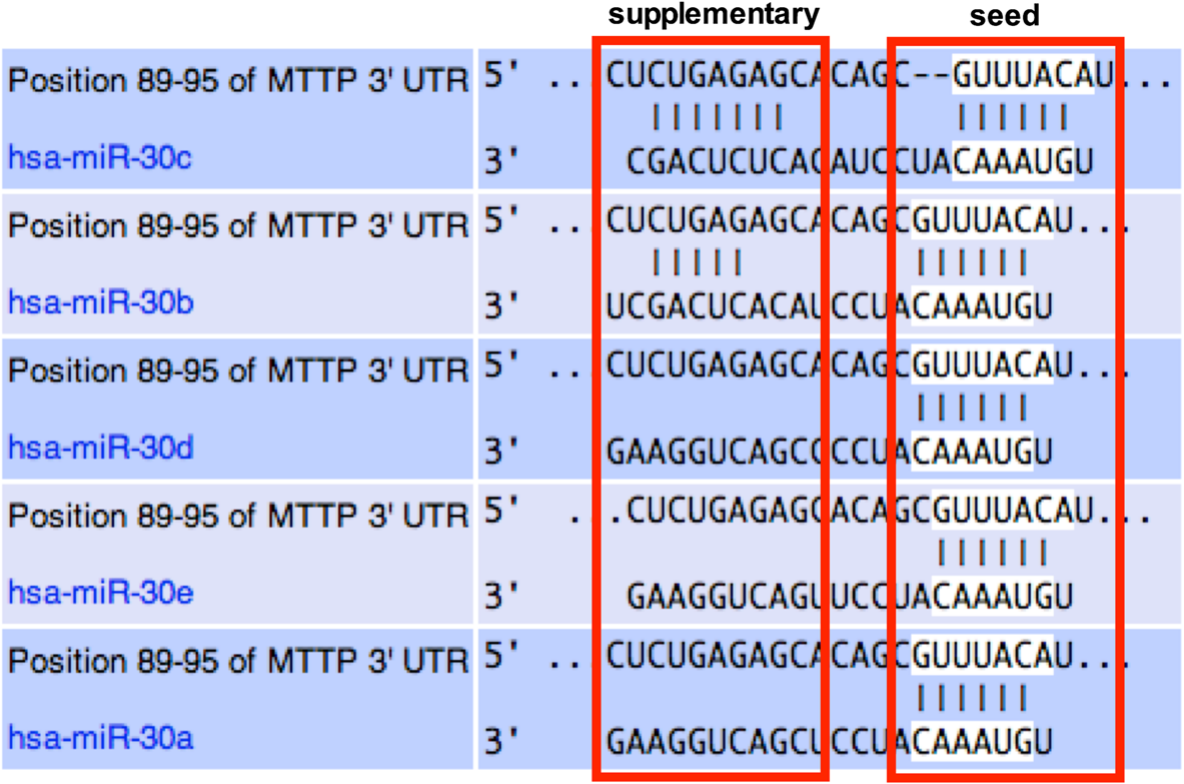
**Predicted interactions between miR-30c family members and 3΄-untranslated region of the MTTP mRNA.** Base pairing between different miR-30 family members and MTTP mRNA was performed using TargetScan. Red rectangles demarcate seed and supplementary interaction sites between miR-30c family members and the MTTP mRNA. Vertical lines indicate base pairings.

We have previously shown that seed sequence is important for the regulation of MTTP by miR-30c as mutations in this sequence in the MTTP mRNA abolishes the ability of miR-30c to reduce MTTP expression [13]. All the miR-30 family members share the same seed sequence and could form similar base pairs with the MTTP mRNA (Figure 1). Yet, only miR-30c regulates MTTP mRNA [13]. Reasons for this specificity are not obvious. Comparative analysis of the miR-30 family members and their possible interactions with MTTP mRNA revealed that these two molecules could form complementary base pairing involving disparate “seed” and “supplementary” sequences resulting in the formation of an intervening asymmetric loop where 8 residues in miR-30c and 6 residues in MTTP mRNA do not form base pairs (Figure 1). Besides miR-30c that forms 7 base pairs, miR-30b can form five base pairs with MTTP mRNA in its supplementary site and yet is unable to regulate MTTP expression [13]. Other miR-30 family members are not predicted to form supplementary base pairs with MTTP mRNA and do not generate a loop (Table 1). Thus, it is possible that specificity of miR-30c might be secondary to its ability to interact with MTTP mRNA at the supplementary site.

### Supplementary site interactions are required for miR-30c to regulate MTTP expression

**Figure 2:**
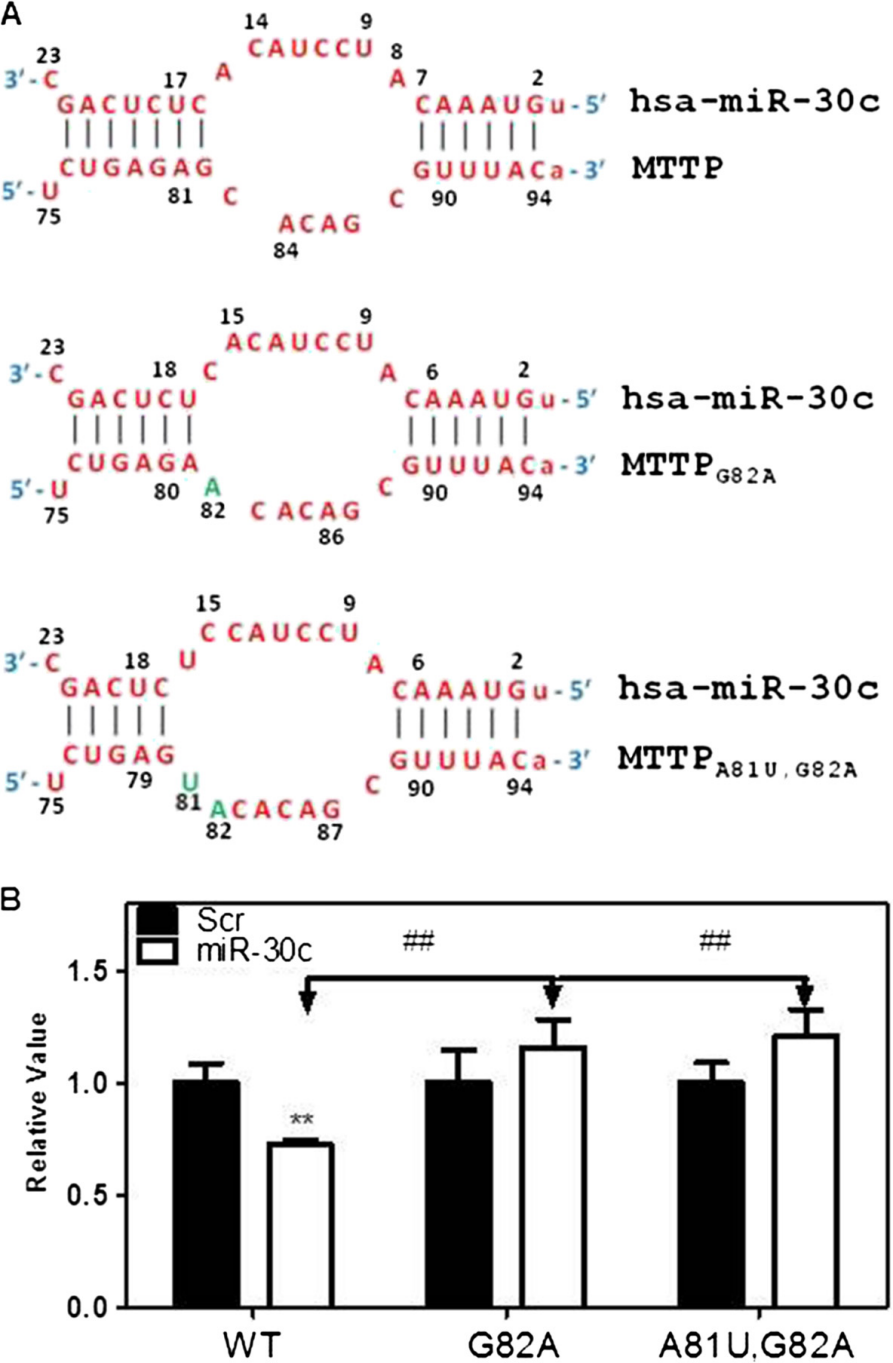
**Supplementary site interactions are critical for the binding of miR-30c to the 3΄-untranslated region of MTTP mRNA. (A)** Interactions between wildtype (MTTP) and miR-30c are shown in the top. Next two structures show mutations introduced in the MTTP 3΄-UTR as green letters and predicted consequences of these mutations on the base pairing with miR-30c. **(B)** Cos-7 cells were first transfected with 50 nM of Scr or miR-30c. Next day cells were transferred to 6-well plates and transfected with 1 μg f wildtype (WT) or different indicated mutants. After 24 h, cells were used to measure luciferase activity. Values in one WT sample was normalized to one and other values were normalized to this value. There were no significant differences amongst different Scr groups. Significant differences between Scr and miR-30c are shown as *. Significant differences between WT and mutant MTTP constructs are shown as #. Mean ± SD, n = 3. **,## p < 0.01.

To determine the importance of number of base pairs in the supplementary site, we used a reporter plasmid (psiCHECK2) with the entire 3′ -UTR of MTTP mRNA subcloned after the stop codon of the *Renilla* luciferase gene. Specific base pairs in the supplementary site that are involved in miR-30c binding were mutated using site-directed mutagenesis. We first designed two mutants, MTTP_G82A_ and MTTP_A81U,G82A_ to reduce the number of base pairs at the supplementary site between miR-30c and MTTP mRNA and increase the size of the loop between seed and supplementary sites (Figure 2A). As anticipated, when miR-30c was transfected into cells expressing luciferase activity with the wildtype (WT) MTTP 3΄-UTR, the luciferase activity was significantly diminished (Figure 2B). However, miR-30c was unable to reduce luciferase activity compared to the Scr control when MTTP_G82A_ and MTTP_A81U,G82A_ mutations were introduced in the 3′-UTR region. Now, there was no significant difference between Scr and miR-30c indicating that miR-30c was unable to interact with the 3΄-UTR of MTTP. These studies indicate that interactions of miR-30c with A81 and G82 residues in the 3′-UTR of MTTP are critical for miR-30c to reduce luciferase activity.

### Increasing base pairing at the supplementary site has no effect on the efficacy of miR-30c

**Figure 3:**
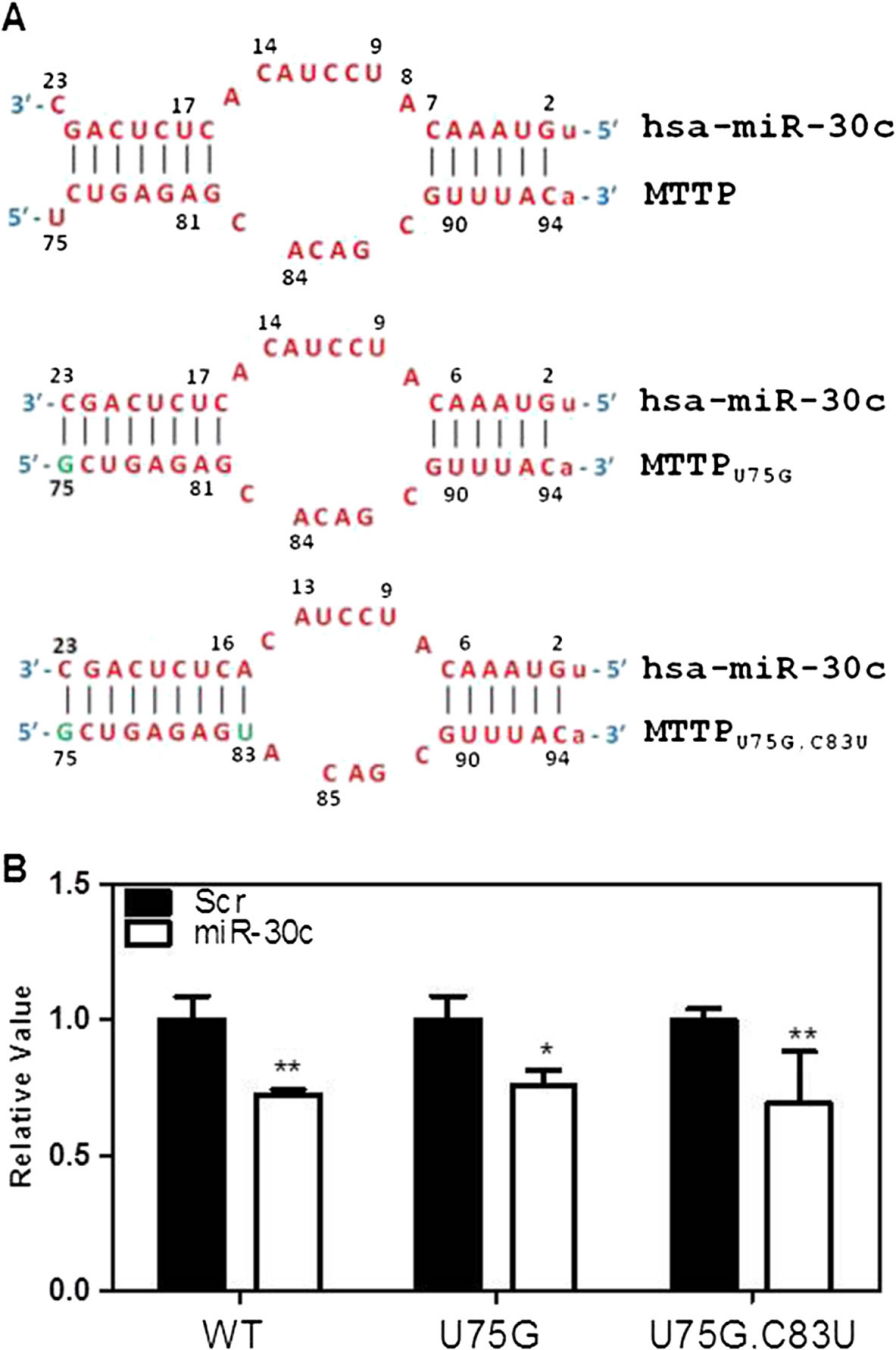
**Increasing base pairings at either ends of the supplementary site has no effect on miR-30c and MTTP 3΄-UTR. (A)** Base pairings between WT and different MTTP mutants and miR-30c are shown. Top row shows interactions between WT MTTP 3΄-UTR and miR-30c. The bottom two rows show mutations (shown as green alphabets) introduced in the MTTP sequence and their consequences on interactions with miR-30c. **(B)** Cos-7 cells were transfected with Scr or miR-30c as described in Methods and Figure 2. Subsequently, cells were transferred to other wells and transfected with luciferase expressing plasmids harboring either WT MTTP 3΄-UTR or different mutants described in Panel A. Significant differences between Scr and miR-30c are shown. Mean ± SD, n = 3. *p < 0.05, **p < 0.01.

After determining that the supplementary site interactions are important, we asked whether increasing the number of base pairs in this region might increase the efficacy of miR-30c. To test this hypothesis, we designed one mutant, MTTP_U75G_ to increase base pairing at the 3΄ end of miR-30c (Figure 3A). Further, an additional mutant, MTTP_U75G,C83U_ was designed to improve binding at both the 3′ and 5′ ends in the supplementary region. The MTTP_U75G_ mutant interacts with 3′-UTR of MTTP involving eight bases while the MTTP_U75G,C83U_ mutant forms nine base pairs at the supplementary site. We anticipated that increasing the number of base pairs in this region will increase the efficacy of miR-30c leading to significantly greater reductions in luciferase activity. Surprisingly, we observed that the MTTP_U75G_ and MTTP_U75G,C83U_ mutants behaved very similar to the wildtype (Figure 3B). Hence, efficacy of miR-30c cannot be improved by increasing single base pairings at either ends of the supplementary site.

### Reducing the loop size between seed and supplementary sites enhances efficacy of miR-30c in reducing MTTP expression

**Figure 4:**
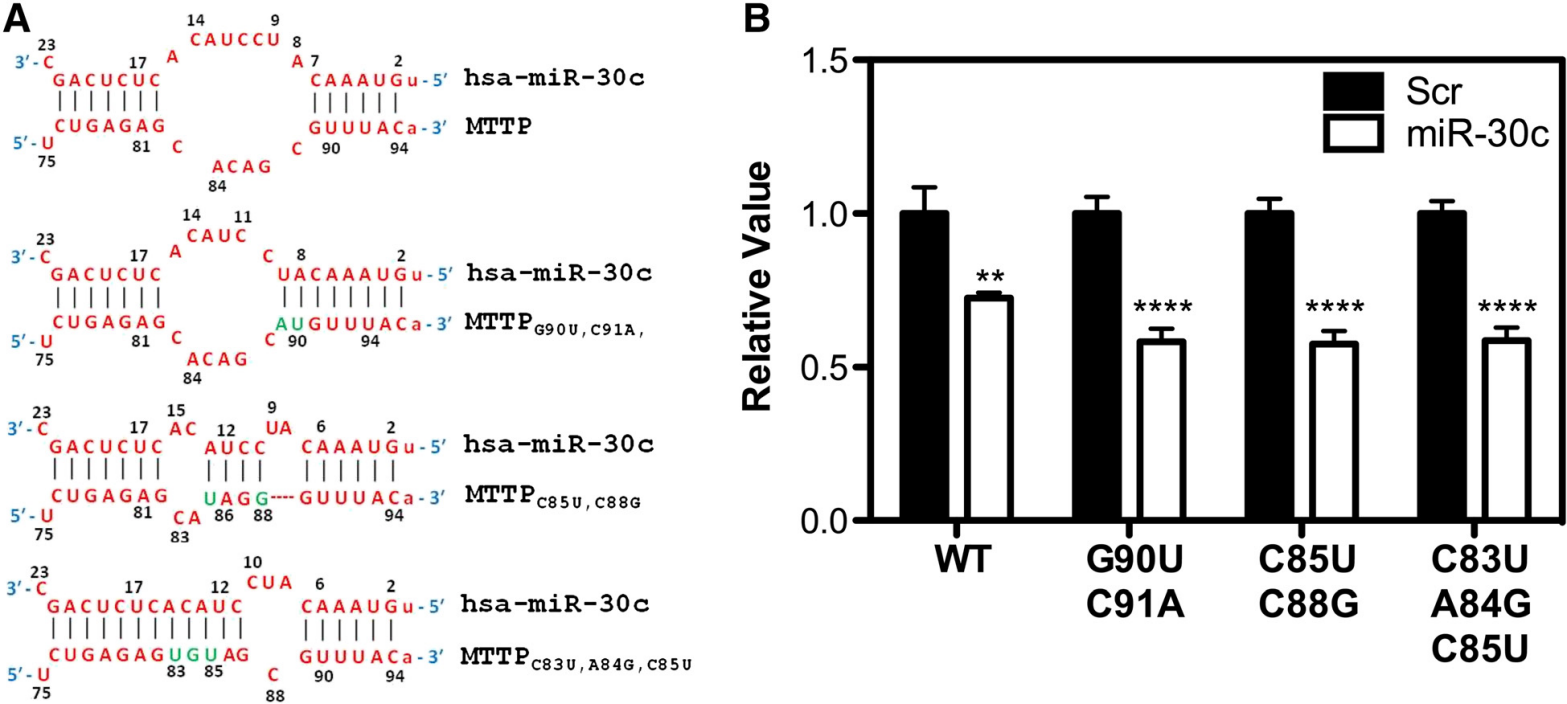
**Increasing base pairing at the seed sequence and in the intervening loop enhances affinity between miR-30c and MTTP 3΄-UTR. (A)** Interactions between WT and different MTTP 3΄-UTR mutants and miR-30c are shown. **(B)** Cos-7 cells were transfected with 50 nM of Scr or miR-30c as described in Figure 2 and Materials and Methods. Cells were then plated in different wells and transfected with plasmids expressing luciferase and containing either WT or mutant MTTP 3΄-UTR. After 17 h, cells were used to measure luciferase activities. Significant differences between Scr and miR-30c are shown. Mean ± SD, n = 3. *p < 0.05, **p < 0.01, ***p < 0.001.

Next, we evaluated importance of the loop present between the seed and supplementary sites for miR-30c/MTTP mRNA interactions. We hypothesized that the asymmetric loop might be important in determining the specificity and efficacy of miR-30c towards MTTP mRNA. Thus, mutations that reduce the size of the loop might result in the loss of miR-30c effect on luciferase expression. To test this, we designed three different mutants that would lead to the formation of varying degrees of shorter loops when miR-30c and MTTP mRNA interact (Figure 4A). The mutant, MTTP_G90U,C91A_ has a slightly smaller loop due to increased interactions at the seed sequence. The MTTP_C85G,C88U_ mutant has mutations within the loop that theoretically reduces the loop and generates two smaller loops. The MTTP_C83U,A84G,C85U_ mutant has mutations that essentially extend the length of the supplementary site interactions to twelve bases with significantly reduced loop size. Surprisingly, all these three mutants showed reduced reporter activity in the presence of miR-30c than wildtype (Figure 4B) suggesting that the mutants respond better to miR-30c than the WT 3΄-UTR of MTTP. This suggests that size of the loop is not critical for proper binding of miR-30c to its target; rather, the closing of the loop improves its efficacy. In future studies, the design of better mimics to target MTTP mRNA may best be suited to close the asymmetrical loop next to the seed and/or supplementary sequences.

**Figure 5:**
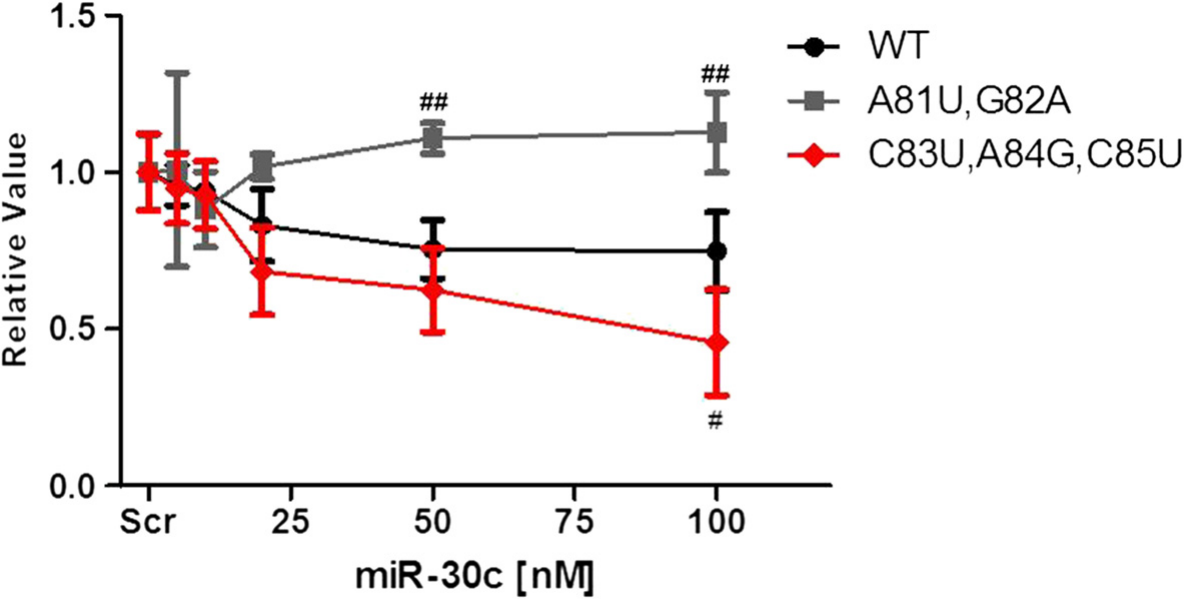
**Effect of different concentrations of mutants on their susceptibility to miR-30c.** Cells were first transfected with different amounts of miR-30c. For control, cells were transfected with 100 nM Scr. Subsequently, cells were transfected with 1 μg of different indicated plasmids. Comparisons were made at each miR-30c concentrations with WT MTTP. Mean ± SD, n = 3. ##, P < 0.01.

The major findings of the above studies were that reducing base pairings in the supplementary site abolish the effect of miR-30c whereas increasing these base pairings by reducing the loop size increases the efficacy. To confirm these studies, we transfected cells with different indicated amounts of plasmids in miR-30c expressing cells and analyzed the luciferase activity (Figure 5). Consistent with Figure 2, mutant MTTP_A81U,G82A_ was unable to reduce luciferase expression at all the concentrations suggesting that these residues are critical for the effect of miR-30c. In contrast, mutant MTTP_C83U,A84G,C85U_ had a tendency to be more effective and reached statistical significance at the highest concentration. These studies establish that interactions at the supplementary site are important for miR-30c to regulate MTTP expression. Further they indicate that it might be possible to increase the efficacy of miR-30c by enhancing base pair interactions between seed and supplementary sites.

## Discussion

We have previously shown that miR-30c lowers both MTTP mRNA and protein levels, but other miR-30 family members do not affect MTTP levels [13]. All the miR-30 family members share a common seed sequence; therefore, it was not clear why only miR-30c regulates MTTP expression. The aim of this study was to find out sequence elements that determine the specificity of miR-30c toward MTTP mRNA. Comparative analysis of the miR-30 family members and their possible interactions with MTTP mRNA revealed that miR-30c could interact with supplementary sequences that are disparate from seed sequences, forming an asymmetric intervening loop where residues in miR-30c and MTTP mRNA do not form any hydrogen bonds. We have previously shown that interaction of miR-30c through seed sequence is necessary to regulate MTTP expression [13]. Here, we studied the importance of supplementary site and intervening loop in determining the specificity between miR-30c and MTTP mRNA. When we reduced the number of base pairs in the supplementary site, miR-30c lost its ability to lower luciferase activity. In particular, MTTP_G82_ was critical for miR-30c binding to the 3′ -UTR of MTTP mRNA. This was further confirmed by the use of MTTP_A81U,G82A_. This mutant was also not responsive to miR-30c. The mutant MTTP_A81U,G82A_ is very similar to miR-30b. The MTTP_A81U,G82A_ mutant forms five base pairs with miR-30c, just like miR-30b. We have previously shown that miR-30b does not affect MTTP expression. Thus, these studies indicate that a minimum of seven base pairs at the supplementary site are required for miR-30c to regulate MTTP expression.

We also examined the 3′ end of the supplementary region to further understand the specific interactions of miR-30c and MTTP. We designed a mutant that targeted the 3΄ end (MTTP_U75G_) and one that targets both the 3′ and 5′ ends (MTTP_U75G,C83U_). The two mutants had no additional effects on miR-30c binding to the 3′ UTR of MTTP. This result was unexpected, as we had predicted that increasing base pairing at the supplementary site would improve binding efficacy. These data suggest that increasing one base pair at the 3′ and 5΄-ends of the supplementary region has no additive benefit in reducing MTTP expression.

Importance of the intervening loop was studied by increasing base pairings at supplementary and seed sequences, and thereby reducing its size. Further, the loop was interrupted by introducing base pairing within the loop. Increasing supplementary interactions to 9 base pairs as in MTTP_C83U,A84G,C85U_ enhanced miR-30c efficacy. Similarly increasing interactions by 2 base pairs at the seed sequence also increased miR-30c efficacy as seen in MTTP_G90U,C91A_. These data indicate that the size of the intervening loop is not critical for the regulation of MTTP expression by miR-30c. This was further supported by the observation that mutant MTTP_C85U,C88G_ that disrupts the loop and creates two small loops appears to act better than the wildtype 3΄-UTR in responding to miR-30c. Thus, increasing base pairing at the seed, supplementary and loop enhances interactions of miR-30c with 3΄-UTR of MTTP mRNA. It is possible that miR-30c mimics with enhanced binding in the intervening loop region might be more potent than miR-30c in reducing MTTP expression.

These studies clearly show that supplementary interactions play a role in determining specificity and enhancing binding between miR-30c and MTTP. Previously, the importance of the interactions at the supplementary site has not been fully appreciated perhaps because of the lack of precise and strong predictive databases that pair miRs with mRNA. Databases that analyze the supplementary region based on free energy, secondary structure, and straightforward base pairing have revealed that residues 13 to 17 may be critical for binding [16,17]. Attempts at analyzing the evolutionary conservation of the supplementary binding sites has not added much to the understanding of the importance of supplementary region [16]. Unlike the analyses of the seed sequence, analyzing the supplementary sequence may be more difficult because of the sheer number of bases (more than seven) and other energy requirements involved in predicting binding sites. Furthermore, maintaining evolutionary conservation in the supplementary region may be more difficult due to energy constraints and the increased number of base pairing involved. Despite the lack of strong predictive databases to study the supplementary region, we have experimentally observed that miR-30c reduces MTTP expression while other members of the miR-30 family do not. Therefore, these studies suggest that experimental validation is required on a case by case basis to establish the importance of supplementary interactions between miRs and their targets.

A caveat of this study is that we only mutated sequences in the 3΄-UTR of MTTP and did not make complementary changes in the miR-30c. This is mainly because we do not have expertise in synthesizing miR mimics and the cost of custom synthesizing miR-30c mimics is prohibitive. However, mutations generated in this study can be used to design better miR-30c mimics that perhaps can be more efficacious than miR-30c. These studies suggest that attempts to design better miR-30c mimics should focus to increase base pairing at the seed sequence and closing the intervening loop between the seed and supplementary sites. These changes might improve affinity between miR-30c and MTTP 3΄-UTR and lower amounts of miR-30c mimics needed to reduce MTTP expression. However, care should be taken to avoid creating a siRNA as MTTP antisense oligonucleotides have not been very useful therapeutic agents [18]. Since bioinformatics algorithms are unavailable that can analyze supplementary site interactions and predict regulation of target mRNAs, there is a need to experimentally determine whether newly designed miR-30c mimics that have higher affinity for MTTP 3΄-UTR would regulate lipid metabolic pathways similar to miR-30c and would not lead to adverse events seen with MTTP inhibition. Nevertheless, we can speculate that increasing interactions between miR-30c and MTTP mRNA will preserve the targeting of additional pathways by miR-30c since these modifications will not alter seed sequence. Improved binding of miR-30c mimics to MTTP mRNA while maintaining the same level of regulation of other lipid synthesis pathways could be the ideal therapeutic modality.

Because of the stringent requirements for the regulation of MTTP by miR-30c, it is possible that mutations in the MTTP 3΄-UTR or in the miR-30c sequences could have subtle effects on lipid metabolism. Hence, it might be useful to determine whether mutations in miR-30c gene(s) and/or MTTP 3΄-UTR could cause hyperlipidemia in humans. However, it should be pointed that mutations in the seed sequence of miR-30c could have unwanted consequences as this might affect interactions with several other targets. But, mutations in the supplementary site might have specific effects on MTP and lipid metabolism.

A word of caution about the use of miR-30c mimics as therapeutic target is that miR-30c, as other miRs, has several other targets. Moreover, miR-30c has been shown to promote adipocyte differentiation [19]. It remains to be determined whether miR-30c therapy will be associated with obesity. Intuitively, this might not occur as high miR-30c concentrations will probably lower intestinal and hepatic lipoprotein production and reduce plasma lipids. Therefore, there will be fewer lipids available for storage in the adipose tissue.

In short, these studies show that the specificity of miR-30c, compared to other members of the miR-30 family, towards MTTP mRNA might be in part due to additional supplementary interactions. Further, these studies showed that intervening loop between seed and supplementary sites is not critical for miR-30c/MTTP mRNA interactions. Additionally, these studies suggest that increasing base pairing in this region might be beneficial in designing a potent miR-30c mimic.

## Abbreviations

apoAI: Apolipoprotein AI; apoB: Apolipoprotein B; MTTP: Microsomal triglyceride transfer protein; miR: microRNA; Scr: Scramble miR; UTR: Untranslated region.

## Competing interests

The authors declare no competing financial interests.

## Authors’ contributions

JS performed and analyzed all the experiments. MMH is the principal investigator who provided financial and idea leadership, designed and discussed experiments, supervised progress and direction of the study, extensively edited and communicated the manuscript. All authors read and approved the final manuscript.
